# Phase II clinical study of valproic acid plus cisplatin and cetuximab in recurrent and/or metastatic squamous cell carcinoma of Head and Neck-V-CHANCE trial

**DOI:** 10.1186/s12885-016-2957-y

**Published:** 2016-11-25

**Authors:** Francesco Caponigro, Elena Di Gennaro, Franco Ionna, Francesco Longo, Corrado Aversa, Ettore Pavone, Maria Grazia Maglione, Massimiliano Di Marzo, Paolo Muto, Ernesta Cavalcanti, Antonella Petrillo, Fabio Sandomenico, Piera Maiolino, Roberta D’Aniello, Gerardo Botti, Rossella De Cecio, Nunzia Simona Losito, Stefania Scala, Annamaria Trotta, Andrea Ilaria Zotti, Francesca Bruzzese, Antonio Daponte, Ester Calogero, Massimo Montano, Monica Pontone, Gianfranco De Feo, Francesco Perri, Alfredo Budillon

**Affiliations:** 1 Head and Neck Medical Oncology Unit, Istituto Nazionale per lo Studio e la Cura dei Tumori, “Fondazione G. Pascale,” IRCCS, Naples, Italy; 2 Experimental Pharmacology Unit, Istituto Nazionale per lo Studio e la Cura dei Tumori, “Fondazione G. Pascale,” IRCCS, Naples, Italy; 3 Head and neck Surgery Unit, Istituto Nazionale per lo Studio e la Cura dei Tumori, “Fondazione G. Pascale,” IRCCS, Naples, Italy; 4 Melanoma and soft tissue Surgery Unit, Istituto Nazionale per lo Studio e la Cura dei Tumori, “Fondazione G. Pascale,” IRCCS, Naples, Italy; 5 Radiotherapy Unit, Istituto Nazionale per lo Studio e la Cura dei Tumori, “Fondazione G. Pascale,” IRCCS, Naples, Italy; 6 Clinical Pathology Unit, Istituto Nazionale per lo Studio e la Cura dei Tumori, “Fondazione G. Pascale,” IRCCS, Naples, Italy; 7 Radiology Unit, Istituto Nazionale per lo Studio e la Cura dei Tumori, “Fondazione G. Pascale,” IRCCS, Naples, Italy; 8 Pharmacy Unit, Istituto Nazionale per lo Studio e la Cura dei Tumori, “Fondazione G. Pascale,” IRCCS, Naples, Italy; 9 Pathology Unit, Istituto Nazionale per lo Studio e la Cura dei Tumori, “Fondazione G. Pascale,” IRCCS, Naples, Italy; 10 Functional Genomics Unit, Istituto Nazionale per lo Studio e la Cura dei Tumori, “Fondazione G. Pascale,” IRCCS, Naples, Italy; 11 Scientific Direction, Istituto Nazionale per lo Studio e la Cura dei Tumori, “Fondazione G. Pascale,” IRCCS, Naples, Italy; 12 Present Address: Medical Oncology Unit, POC SS Annunziata, Taranto, Italy

**Keywords:** Cetuximab, Cisplatin, Head and Neck cancer, Histone deacetylase inhibitor, Valproic acid

## Abstract

**Background:**

Recurrent/metastatic squamous cell carcinoma of the head and neck (SCCHN) has a poor prognosis and the combination of cisplatin and cetuximab, with or without 5-fluorouracil, is the gold standard treatment in this stage. Thus, the concomitant use of novel compounds represents a critical strategy to improve treatment results. Histone deacetylase inhibitors (HDACi) enhance the activity of several anticancer drugs including cisplatin and anti-Epidermal Growth Factor Receptor (anti-EGFR) compounds. Preclinical studies in models have shown that vorinostat is able to down regulate Epidermal Growth Factor Receptor (EGFR) expression and to revert epithelial to mesenchimal transition (EMT). Due to its histone deacetylase (HDAC) inhibiting activity and its safe use as a chronic therapy for epileptic disorders, valproic acid (VPA) has been considered a good candidate for anticancer therapy. A reasonable option may be to employ the combination of cisplatin, cetuximab and VPA in recurrent/metastatic SCCHN taking advantage of the possible positive interaction between histone deacetylase inhibitors, cisplatin and/or anti-EGFR.

**Method/Design:**

V-CHANCE is a phase 2 clinical trial evaluating, in patients with recurrent/metastatic squamous cell carcinoma of the head and neck never treated with first-line chemotherapy, the concomitant standard administration of cisplatin (on day 1, every 3 weeks) and cetuximab (on day 1, weekly), in combination with oral VPA given daily from day −14 with a titration strategy in each patient (target serum level of 50–100 μg/ml). Primary end point is the objective response rate measured according to Response Evaluation Criteria in Solid Tumors (RECIST). Sample size, calculated according to Simon 2 stage minimax design will include 21 patients in the first stage with upper limit for rejection being 8 responses, and 39 patients in the second stage, with upper limit for rejection being 18 responses. Secondary endpoints are time to progression, duration of response, overall survival, safety.

Objectives of the translational study are the evaluation on tumor samples of markers of treatment efficacy/resistance (i.e. γH2AX, p21/WAF, RAD51, XRCC1, EGFR, p-EGFR, Ki-67) and specific markers of VPA HDAC inhibitory activity (histones and proteins acetylation, Histone deacetylase isoforms) as well as valproate test, histones and proteins acetylation of peripheral blood mononuclear cell, tested on blood samples at baseline and at different time points during treatment.

**Discussion:**

Overall, this study could provide a less toxic and more effective first-line chemotherapy regimen in patients with recurrent/metastatic squamous cell carcinoma of the head and neck by demonstrating the feasibility and efficacy of cisplatin/cetuximab plus valproic acid. Moreover, correlative studies could help to identify responder patients, and will add insights in the mechanism of the synergistic interaction between these agents.

**EudraCT Number:**

2014-001523-69

**Trial registration:**

ClinicalTrials.gov number, NCT02624128

## Background

### Histone deacetylases inhibitors (HDACi) as anticancer agents

Epigenetic alterations, such as hypoacetylation of histones, play an important role in initiation and progression of several cancers, including squamous cell carcinoma of the head and neck (SCCHN). Since epigenetic alterations are dynamic and generally reversible, epigenetic manipulation has emerged as an attractive novel anticancer treatment. Histone Deacetylase inhibitors (HDACi) are emerging epigenetic antitumor agents [1]. A large number of HDACi are currently in clinical development as anticancer agents, and three (vorinostat, romidepsin and belinostat) have been approved by the US FDA for the treatment of cutaneous T-cell lymphoma [2, 3],^1^ while panobinostat is the first HDAC inhibitor approved to treat multiple myeloma in combination with the proteasome inhibitor bortezomib and dexamethasone, in patients who have received at least two prior standard therapies.^2^ Our group and many others have demonstrated the synergistic antitumor activity of HDACi in combination with several chemotherapeutics and molecular targeted agents, including cisplatin and anti-epidermal growth factor receptor (EGFR) agents [4–7]. In details, we have recently demonstrated that the HDACi vorinostat, in combination with the EGFR-tyrosine kinase inhibitor gefitinib, induced synergistic inhibition of proliferation, migration and invasion as well as induction of apoptosis, in preclinical models of SCCHN, including cancer cell lines resistant to gefitinib and characterized by mesenchymal markers and phenotype. The mechanism of the synergistic interaction is related to the ability of vorinostat to modulate the expression and the activity of ErbB receptors (EGFR, ErbB2 and ErbB3) and to reverse the epithelial mesenchymal transition (EMT) in gefitinib-resistant cells [5].

### Valproic acid: preclinical and clinical studies

Valproic acid (VPA), an anticonvulsant clinically effective also as a mood stabilizer in the treatment of maniac depression (bipolar affective disorder) has HDAC inhibitory activity and anticancer properties with good safety profile compared with other HDACi [8, 9].

The recommended values of serum concentrations for epilepsy treatment are in the 50–100 μg/ml range. Phase-1/2 studies in several malignancies showed that VPA, either as a monotherapy or in combination with other agents, was well tolerated with some encouraging responses. In monotherapy at oral doses between 20 and 60 mg/kg VPA inhibit deacetylase activity in solid tumors [10]. VPA oral doses of 30 mg/kg daily in combination with the demethylating agent hydralazine, doxorubicin and cyclophosphamide, as neoadjuvant therapy in locally advanced breast cancer patients, was safe and tumor responses appeared higher as compared with historical controls; HDAC inhibition was demonstrated in the peripheral blood of the patients, with a mean plasma concentration of 87.5 μg/ml [11]. In another phase I/II trial, VPA in combination with chemotherapy (FEC100) for patients with solid tumors, demonstrated a maximum tolerated dose (MTD) of 140 mg/kg/day with nine patients achieving a partial response. During the second part of the study, a disease-specific cohort breast cancer patients were treated with VPA 120 mg/kg/day plus FEC100 regimen; 9 out of 14 patients responded and somnolence was the most noted VPA-related adverse effect [12]. Notably, VPA crosses the blood–brain barrier, and can be safely utilized for long time frames. All the above characteristics point to VPA as an appealing drug for clinical studies.

VPA is one of the most studied HDACi in combination therapy with platinum-based drugs in many cancer cell models including SCCHN [8]. Currently valproate is being evaluated in combination with cisplatin in a phase 2 clinical trial in refractory and recurrent mesothelioma patients [13]. We have recently launched a Phase ½ clinical study of VPA in combination with capecitabine and short-course radiotherapy as preoperative treatment in locally advanced rectal cancer patients (EudraCT Number: 2012-002831-28) [14].

## VPA safety and cardiac toxicity

Common toxicities demonstrated by almost all HDAC inhibitors including thrombocytopenia, neutropenia, diarrhea, nausea, vomiting and fatigue, were not reported for VPA treatment, being somnolence the only dose-limiting adverse effect. Several additional mild and transient side effects were described for VPA but most of them were related with its chronic use [15]. In details, weight gain, changes in serum triglycerides, cholesterol and fast glucose were described, as well as some dermatological effects such as stomatitis, cutaneous leukocytoclastic vasculitis and psoriasis-like eruption. Due to its direct neurological action some rare neurological side effects were also reported including encephalopathy, VPA-induced parkinsonism, and hyperammonemia in the absence of liver failure. Hepatotoxicity has been also reported particularly in young children and in the presence of hepatic disorders. Finally, one study reported the increased risk of aplastic anemia after the use of VPA, but opposite evidences reported VPA as a potent activator of erythropoiesis in epileptic patients.

Extensive studies have been performed to determine whether HDAC inhibitors are associated with cardiac toxicities [2, 16–19]. In a phase I trial of VPA in combination with epirubicin, a grade 2 QTc prolongation was reported in eight patients (18%), and a grade 3 QTc prolongation was seen in two patients (5%); these events occurred predominantly on day 1 of VPA treatment. QTc prolongations were associated with serum potassium levels less than 4.0 mmol/L and were resolved in all patients with appropriate potassium and magnesium supplementation [20].

## Rationale

### Combination of an HDAC inhibitor with anti EGFR agent and platinum derivatives in SCCHN

SCCHN accounts for 6–7% of all malignancies, representing the fifth most common tumor worldwide. About 50% of the patients who have been treated for an early stage or a locally advanced disease, will experience a recurrent and/or metastatic disease. Albeit several therapy improvements have been registered in the last years, the prognosis of patients with recurrent/metastatic disease remains poor, particularly for those with the traditional risk factors of tobacco and/or alcohol use as compared with patients with human papilloma virus (HPV)-driven disease. The application of targeted therapeutics in SCCHN has been disappointing to date as compared to other cancer types. A number of additional therapeutic targets have been proposed for SCCHN based on recent genomic discovery studies and preclinical studies but none have been confirmed so far in clinical studies [21]. Cisplatin is the mainstay of combinatory treatment for several solid tumors, including unresectable and recurrent/metastatic SCCHN. This drug is often associated with the antimetabolite 5-fluorouracil (5FU), showing a good anti-cancer response but also many toxic effects as well as treatment-resistance. Overexpression of EGFR and of its ligands TGF-α or EGF has been observed in about 90% of SCCHN specimens, with the exception of HPV-positive tumors, and correlates with poor disease-free and overall survival, an increased risk of disease recurrence and metastasis, and resistance to chemotherapy, including cisplatin, and radiotherapy [22]. Interestingly, it was previously reported that EGFR over-expression has an important role in the induction of resistance to cisplatin treatment. In details, it has been demonstrated that tumor cells treated with cisplatin show increased EGFR activation which can be considered a survival response to the treatment [23].

Therefore, EGFR is considered to be an excellent target for this disease, and the anti-EGFR monoclonal antibody cetuximab (CTX), although yielding only modest clinical activity in monotherapy, is the only targeted therapy approved for the treatment of SCCHN in patients with locally advanced tumors in association with radiotherapy and in patients with recurrent or metastatic disease in combination with cisplatin-based chemotherapy [22, 24–26].

In addition, the transdifferentiation of epithelial cells into mesenchymal cells, known as EMT, a key process required during embryonic development and associated with the development of invasive cancer [27], seems also to play a role in the resistance to EGFR TKIs in several tumors, including SCCHN [28]. Moreover, a mesenchymal-enriched subtype represents a distinct type of SCCHN with a defined recurrence-free survival prognosis.

Our group and many others have demonstrated the synergistic antitumor activity of HDACi in combination with a large number of structurally different anticancer agents, among which cisplatin and anti-EGFR agents [4–7]. Our group has recently demonstrated that the HDACi vorinostat, in combination with the EGFR-tyrosine kinase inhibitor gefitinib, induced synergistic inhibition of proliferation, migration and invasion as well as induction of apoptosis, in preclinical models of SCCHN and NSCLC, including cancer cell lines resistant to gefitinib and characterized by mesenchymal markers and phenotype. The mechanism of the synergistic interaction is related to the ability of vorinostat to modulate the expression and the activity of ErbB receptors (EGFR, ErbB2 and ErbB3), to reverse EMT, and/or to alter redox homeostasis in gefitinib-resistant cells [5, 7, 29].

VPA is one of the most studied HDACi in combination therapy with platinum-based drugs in many cancer cell models including SCCHN [8]. Currently valproate is being evaluated in combination with cisplatin in a phase II clinical trial in refractory and recurrent mesothelioma patients [13].

### Biomarkers study

SCCHN, given the relative accessibility of tumor to sample, is an ideal cancer type to assess the efficacy of new therapeutic approach with biopsy samples taken before and during treatment to identify biomarkers of response and resistance.

VPA serum levels was correlated in several studies with histone acetylation in tumor samples and in PBMC and was also linked to baseline expression of HDAC isoforms. In oral tongue cancer (generally HPV-negative) it has been demonstrated that HDAC1 and 2 are overexpressed and associated with significantly shorter progression-free survival (PFS) [30]. Moreover, in several clinical studies the measurement of histone acetylation on PBMC has been studied as a surrogate marker of HDACi activity; however, in most cases this measurement has not been successfully linked to clinical outcome. Recently Yardley et al., have analyzed protein lysine acetylation in pre- and post-treatment samples collected in a subset of 49 breast cancer patients treated with the combination of the HDACi entinostat plus exemestane demonstrating that hyperacetylation of protein lysines in PBMC was associated with improved clinical outcome, as shown by the prolonged PFS in hyperacetylators versus low acetylators [31]. Elevated levels of protein lysine acetylation maintained in certain patients despite entinostat levels at or below the level of detection at the time of sampling seem to reflect the durability and potency of the pharmacodynamic effects that low sustained concentrations of entinostat can elicit. Biomarker studies in clinical trials have shown that, besides histone hyperacetylation, the major effects of HDAC treatment in solid tumors were p21 overexpression and Ki-67/MIB-1 downregulation, two features typically related with cell differentiation and growth arrest. As mentioned above, HDAC inhibitor can sensitize cancer cells to cisplatin by different mechanisms including the regulation of the expression of DNA repair genes such as RAD51 [32] and the downregulation of antiapoptotic genes such as BCL2 and XIAP [8]. There are evidences that cancer with moderate expression of EGFR are more sensitive to CTX compared with those that express very high levels of EGFR [33]. Preclinical study showed CTX resistant cell line express not only increase in EGFR but in also other members of the same family and in particular HER2 and HER3 as well as MET. HER4 has been observed overexpressed in some HNSCC cancer cell lines where it is associated with higher proliferative rate [34]. It has been observed that MET expression represent an independent predictor of reduced disease-free and overall survival in HNSCC patients [35]. Even more compelling are data that correlate MET expression and radiation [35], cisplatin [36, 37] and CTX [38] in SCCHN. The majority of SCCHN (>90%) overexpress the epidermal growth factor receptor (EGFR or HER1) HER1, which correlates with a poor prognosis and overall resistance to therapy. Immunotherapy with the EGFR-specific IgG1 mAb, CTX, significantly improves survival of SCCHN patients with advanced or metastatic disease [25]. The available evidence is consistent with the possibility that the beneficial effects of CTX administration on the clinical course of the disease reflect both inhibition of EGFR tyrosine phosphorylation and triggering of antibody-dependent, cell-mediated cytotoxicity (ADCC) of SCCHN cells [39]. Binding of CTX to the EGFR leads to internalization and degradation of the antibody–receptor complex and down-regulation of EGFR expression [33]. Also CTX can activate immune cells that bear receptors for the Fc (constant portion) of IgG such as natural killer (NK) cells. NK cells have an activating Fc receptor for IgG (FcγRIIIa), which mediates Ab dependent cellular cytotoxicity (ADCC) and enhances production of interferon-γ (IFN-γ) in response to Ab-coated targets [40, 41]. We previously demonstrated that FcγRIIIa polymorphisms were significantly associated with response to anti-EGFR-based therapy in 49 colorectal cancer patients with KRAS wt tumors, The results suggested that prognosis is particularly unfavorable for patients carrying the FcγRIIIa-158F/F genotype [42].

## Methods/Design

V-CHANCE is a phase 2 trial exploring the feasibility and the activity of VPA in combination with the standard Cisplatin-Cetuximab in recurrent/metastatic SCCHN never treated with first-line chemotherapy patients. The study includes an explorative analysis of the potential prognostic or predictive role of several biomarkers with the aim of improving the knowledge of the mechanisms by which VPA enhances chemotherapy effect and of identifying early predictors of treatment response/resistance.

### Objectives

The primary objective of the study is to assess whether VPA, given concomitantly with the conventional cisplatin-cetuximab regimen, can improve treatment activity (in terms of objective response rate) in patients with recurrent/metastatic SCCHN.

Secondary objectives are response duration, time to progression, overall survival, safety.

A translational study is also planned with several objectives: (a) to compare the expression of several biomarkers (p21/WAF, p16/INK4 and Ki-67/MIB-1, histones and proteins acetylation (H&P-Ac), HDAC isoforms) in the tumor and normal mucosa, to evaluate the tumor expression of markers of treatment efficacy/resistance (pEGFR, MET, RAD51, XRCC1, Bcl2 and XIAP γH2AX, VEGF). (b) to evaluate Histones and proteins acetylation on PBMC as additional surrogate pharmacodynamic markers of VPA activity at different time points during and after treatment. (c) to ensure achievement of the target serum level range, performing valproate test, and to compare it with histones and proteins acetylation.

### Ethical aspects

The procedures set out in this study protocol are designed to ensure that the principles of the Good Clinical Practice guidelines of the International Conference on Harmonization (ICH) and the Declaration of Helsinki are respected in the conduct, evaluation and documentation of this study. The study was approved by the Independent Ethical Committee (CEI) of the National Cancer Institute of Naples, Italy (Clearence obtained with prot. N. CEI/304/14, 17.07.2014). Patients provide written informed consent for participating in the study and for allowing to collect tissue and blood samples.

### Study design

V-CHANCE is a phase 2 study performed in patients with recurrent/metastatic SCCHN never treated with first-line chemotherapy. Patients will be treated with cisplatin (75 mg/m^2^ on day 1 to be repeated every 21 days), CTX (loading dose of 400 mg/m^2^ followed by a maintenance dose of 250 mg/m^2^ to be repeated weekly) and VPA (increasing oral doses, from 500 mg/day on day −14 until a full dose of 1500 mg at day 1, with a titration strategy in a patient for a target serum level range of 50–100 μg/ml). RECIST criteria version 1.1 will be employed with the aim to determine the response rate. In particular, complete responses (CR) will be defined as the total disappearance of all target lesions; partial response (PR) will be observed when the sum of largest diameters of the target lesions will decrease by at least 30%. A 20% increase in the sum of diameter of target lesions will qualify as progressive disease (PD). Stable disease will be defined as neither sufficient shrinkage to qualify for PR nor sufficient increase to qualify for PD. Overall response rate (ORR) will be calculated as the sum of CRs and PRs; while disease control rate (DCR) will correspond to the sum of PS, CRs and SDs).

#### Sample size calculation

Simon 2 stage minimax design will be used for this trial. First stage sample size will include 21 patients and the upper limit for first stage rejection of drugs will be eight patients. Maximum sample size will be 39 patients with the upper limit for second stage rejection being 18 patients. Patients have to be enrolled with the aim to distinguish between the null and alternative hypotheses, with a significance level of 0.05 and a power of 80%.

### Patient selection criteria

#### Inclusion criteria

Patients ≥ 18 years, diagnosed, histologically or cytologically, with squamous cell carcinoma of head and neck (except nasopharynx) will be admitted in the study. Patients have to have first-line recurrent and/or metastatic disease and no prior chemotherapy except for chemo-radiation or induction chemotherapy followed by local treatment given in the context of a curative strategy. ECOG performance status ≤1 at study entry, a life expectancy > 3 months, normal bone marrow reserve, hepatic function, renal function, cardiac function are additional inclusion criteria; effective contraception is mandatory for both male and female patients if the risk of conception exists; a written informed consent has to be signed.

#### Exclusion criteria

Main exclusion criteria are the following: Concomitant treatment with other experimental drugs; brain metastases (CT scan or MRI required only in case of clinical suspicion of CNS metastases); non-squamous cell histology; any concurrent malignancy (patient with a previous malignancy but without evidence of disease for 5 years will be allowed to enter the trial); history of myocardial infarction within the last 12 months; significant cardiovascular comorbidity; patients with long QT-syndrome, or QTc interval duration > 480 msec, or concomitant medication with drugs prolonging QTc; known or suspected hypersensitivity to any of the study drugs; patient who have had prior treatment with an HDAC inhibitor and patients who have received compounds with HDAC inhibitor-like activity, such as VPA; major surgical procedure, within 28 days prior to study treatment start; patients who cannot take oral medication, who require intravenous feeding, have had prior surgical procedures affecting absorption, or have active peptic ulcer disease; pregnant or lactating women and sexually active males and females (of childbearing potential) unwilling to practice contraception during the study.

### Treatment plan

Treatment with VPA includes a titration strategy applied in each patient looking for a serum concentration that is considered useful to produce the desired synergistic effect with cisplatin and CTX. Treatment will be administrated orally starting at day −14 with a 500 mg slow releasing tablet at evening. Thereafter, the dose will be increased also using 300 mg tablets (Table 1).

**Table 1:** Valproic acid dose titration

Days	Morning dose (mg)	Afternoon dose (mg)	Evening dose (mg)
−14 and −13	0	0	500
−12 and −11	300	0	500
−10 and −9	500	0	500
−8 and −7	500	300	500
−6 and −5	500	500	500
−4 and −3	500	500	500
−2 and −1	500	500	500

The interval between each dose will be 12 h from the −14 day to −9 day and it will be 8 h from −8 day

In the morning of day −4, within 2 h after taking the morning dose, serum level of VPA will be checked using a commercially available valproate test, and will be adjusted depending on the reached steady level. The target serum level range will be 50–100 μg/ml which represents the recommended values for the treatment of epilepsy. At any time, in case of grade 2 somnolence or fatigue the VPA dose will be reduced by 200 mg/day steps up to reaching grade ≤1 independently of the actual serum level. In case of grade ≥3 somnolence or fatigue VPA will be definitely discontinued. In case of asymptomatic QTc prolongation development (QTc >500 ms, or QT prolongation >600 ms,) VPA has to be interrupted. Electrolytes and concomitant medications have to be checked and corrected. ECG has to be repeated after 24 h. If the event is resolved, treatment with VPA can be resumed but the dose will be reduced by −200 mg/day; on the contrary, if QT prolongation is confirmed VPA has to be interrupted [43, 44]. In case of symptomatic QTc prolongation development (QTc > 500 ms or QT prolongation > 600 ms,) and association with symptoms suggestive of a ventricular tachyarrhythmia, VPA has to be interrupted. At day 1 cisplatin at dose of 75 mg/m2 given every 3 weeks and CTX at induction dose of 400 mg/m2 followed by maintenance doses of 250 mg/m2 given weekly, will be administered. Adequate intravenous hydration will be required prior and after cisplatin administration. Antiemetic prophylaxis with dexamethasone and palonosetron before cisplatin will be also administered. Toxicity due to cisplatin administration may be managed by symptomatic treatment, dose interruptions and dose adjustment. Once the dose has been reduced it should not be increased at a later time. Doses of cisplatin omitted for toxicity are not replaced or restored. At the time of recycling, blood tests have to be normal (Absolute Neutrophil Count 1.5 × 10^9^/L, platelets 100 × 10^9^/L). If lower values, or at least grade 2 non-hematologic toxicities, are detected, treatment will be interrupted and restored when toxicity is back to grade 1, treatment will be restarted at the same drug dosage. If at any time during a chemotherapy cycle febrile neutropenia, grade 4 neutropenia, grade 4 thrombocytopenia, non-hematologic grade 3 toxicities occur, the subsequent chemotherapy doses will be administered with 50% of the initial dose. No primary prophylaxis with G-CSF is allowed, but secondary prophylaxis is allowed.

### Assessment and procedures

**Fig. 1:**
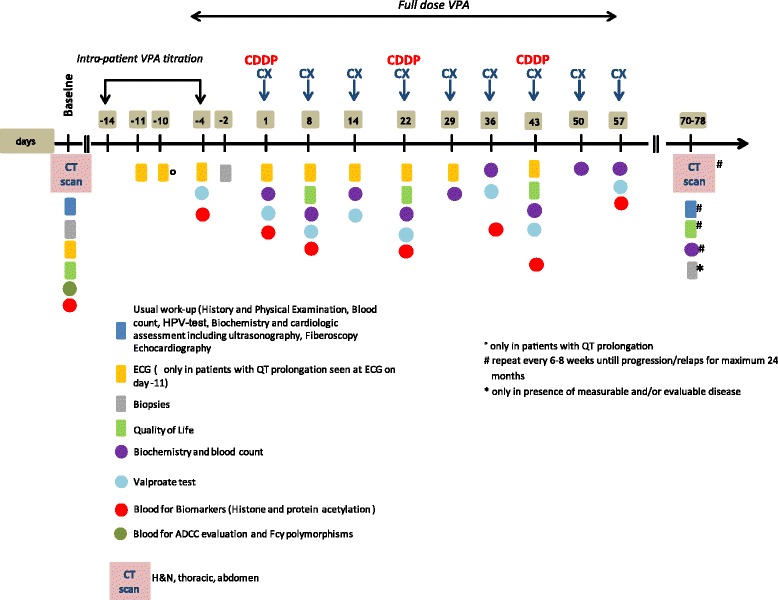
Schematic timeline of study procedures. Note. History and physical examination, blood count, biochemistry will be repeated weekly during treatment

Assessment and procedures, including those for exploratory objectives (see below) are illustrated in Fig. 1. Briefly, baseline procedures will include: HPV-test, full laboratory tests evaluation, cardiologic assessment including ultrasonography, fiberoscopy and Computed Tomography scan of head, neck, thorax and abdomen. Other tests may be performed at the researcher’s discretion.

Each treatment cycle will last 21 days, including administration of cisplatin and CTX on day 1, only CTX repeated on days 8 and 15 and VPA will be given orally throughout the entire cycle. A complete physical exam and a complete serum evaluation of blood cell count, electrolytes, renal and liver function will be performed weekly. Serum concentration of VPA will be assessed every 2 weeks. A complete restaging will be performed after three cycles of chemotherapy and will consist of Computed Tomography scan of head, neck, thorax and abdomen within 21 days from the last chemotherapy administration, a new fibroscopy, a second evaluation of H3 acetylation on blood mononuclear cells peripheral extracted from a peripheral blood sample, a second tumor biopsy in which immunohistochemical assay will be done in order to evaluate marker modification upon treatment. The last one will be performed at the end of treatment (after 3 or 6 cycles) only in presence of measurable and/or evaluable disease.

#### Toxicity evaluation criteria

Acute toxicity will be assessed weekly with clinical examination and blood tests using Common Toxicity Criteria for Adverse Events (CTCAE) of the National Cancer Institute, version 4.0, June 14, 2010.

#### Response evaluation criteria

Response is assessed after 3 cycles. In case of CR, PR, SD, 3 additional cycles will be given. Patients will receive a maximum of 6 cycles. Follow-up tests were carried out every 2 months, until progression.

### Biomarkers

Tumor biopsy and normal mucosa will be collected at baseline (before starting VPA treatment) and possibly within the diagnostic biopsy) at day −2, before starting chemotherapy, and after 3 or 6 cycles of treatment at the first or second evaluation only in presence of measurable and/or evaluable disease. The following markers will be measured: p21/WAF, p16/INK4 and Ki-67/MIB-1, histones and proteins acetylation, as surrogate pharmacodynamic markers of VPA activity on tumor; HDAC isoforms, only at baseline, as potential predictive markers of VPA activity. The tumor expression of all the markers at baseline will be compared with normal mucosa expression and with the tumor expression after treatment. Moreover, at baseline, at day −2 and eventually after 3–6 cycles (see above), EGFR, p-EGFR, MET, RAD51, XRCC1, γH2AX, will be measured as markers of treatment efficacy/resistance evaluated by real-time PCR with the specific primers and probes or by immunohistochemistry. Peripheral blood samples will be collected at baseline, at day −4, 1, 8, at the end of every cycle, at day 22 and at the end of treatment. Histones and proteins acetylation on PBMC will be done as additional surrogate pharmacodynamic markers of VPA activity at different time points during and after treatment. Valproate test will be performed to ensure achievement of the target serum level range and to compare it with histones and proteins acetylation.

Moreover, the CTX induced ADCC activity will be evaluated at baseline by an in vitro assay according to the previoulsy described methods [42] on PBMC and results will be correlated with polymorphisms of FcyRIIa- H131R and the FcyRIIIa-V158F.

### Statistical analysis

The overall response rate (ORR) will be calculated with 95% confidence interval. Time to progression, duration of response and overall survival will be calculated from the first treatment day until the day of event occurrence (for OS the date of death or the date of termination of the trial for patients alive at the time end of the study, or the date of the last follow-up information available for patients lost before the trial end date). Kaplan-Meier methods will be used to estimate all time to event endpoints. For each patient and type of toxicity, the worst degree suffered during the treatment will be described.

Due to the small sample size, statistical analysis of biomarkers data will be conducted with the aim of hypothesis generation. First of all, a complete description of data from biological and pharmacogenomic studies will be done. For biomarkers that might change over time as a consequence of treatment, levels before and after treatment will be compared with appropriate statistical tests, based on the type of data. Serum levels of VPA throughout treatment will be described and compared between different acetylator phenotypes, with appropriate statistical tests. *P* values ≤0.05 will be considered significant, and no adjustment is planned for multiple comparisons due to the exploratory nature of the analysis.

### Quality assurance and data collection procedures

The procedures set out in this study protocol are designed to ensure that the principles of the Good Clinical Practice guidelines of the International Conference on Harmonization (ICH) and the Declaration of Helsinki are respected in the conduct, evaluation and documentation of this study. Patient registration and data collection are centralized at the National Cancer Institute of Naples. Biological analyses are centralized at the Experimental Pharmacology Unit of the NCI of Naples.

## Discussion

In spite of improvements in the treatment of squamous cell carcinoma of the head and neck, the prognosis of patients with recurrent/metastatic disease remains poor. The goal of V-CHANCE study is to demonstrate the feasibility and efficacy of cisplatin/CTX plus VPA to provide a less toxic and more effective first line chemotherapy regimen in patients with R/M SCCHN. The choice of VPA as additional drug in patients treated with cisplatin and CTX should provide a three- drug regimen whose toxicity should not exceed that of the standard two-drug regimen. A new three-drug regimen to be considered less toxic than the 5-fluorouracil-containing other standard, adding a safe and low cost generic drug with HDACi activity such as VPA to the doublet cisplatin-CTX.

Furthermore, the correlative studies could identify potential appealing prognostic/predictive biomarkers of toxicity and efficacy adding also new insight in the mechanism of interaction between VPA, cisplatin and CTX.

Overall, this study is basically aimed at finding out new standards of care in recurrent/metastatic SCCHN.

## Abbreviations

5FU: 5-fluorouracil
ADCC: Antibody dependent cellular cytotoxicity
CNS: Central Nervous System
CR: Complete responses
CT: Computed tomography
CTCAE: Common toxicity criteria for adverse events
CTX: Cetuximab
DCR: Disease control rate
DLT: Dose limiting toxicity
ECG: Electrocardiogram
ECOG: Eastern Cooperative Oncology Group
EDTA: Etilendiamminotetraacetic acid
EGFR: Epidermal growth factor receptor
EMT: Epithelial to mesenchimal transition
FDA: Food and Drug Administration
FEC100: 5-fluorouracil epirubicin, and cyclophosphamide
GCP: Good clinical practice
HDACi: Histone deacetylase inhibitors
HPV: Human papillomavirus
ICH: International Conference on Harmonization
MRI: Magnetic resonance imaging
MTD: Maximum tolerated dose
ORR: Overall response rate
PBMC: Peripheral blood mononuclear cells
PD: Progressive disease
PFS: Progression free survival
PR: Partial response
PS: Performance status
PVCs: Premature ventricular contractions
R/M: Recurrent/Metastatic
RECIST: Response evaluation criteria in solid tumors
SCCHN: Squamous cell carcinoma of the head and neck
SVC: Superior vena cava
TGF- α: Tansforming growth factor alpha
VOR: Vorinostat
VPA: Valproic acid

## Footnotes

1
http://www.fda.gov/newsevents/newsroom/pressannouncements/ucm403929.htm

2
http://www.fda.gov/NewsEvents/Newsroom/PressAnnouncements/ucm435296.htm
